# Supplementary material for: Complexome profiling of the Chlamydomonas psb28 mutant reveals TEF5 as an early PSII assembly factor
Source: Plant Cell. 2025 Mar 17;37(6):koaf055. doi: 10.1093/plcell/koaf055 (PMC12164590; doi:10.1093/plcell/koaf055)
Supplement: koaf055_Supplementary_Data [file koaf055_supplementary_data.zip › psb28_interactive.zip!/plots/intensity/qualityPlots/peptides/_Cre12.g549950.t1.2.html]

### Cre12.g549950.t1.2

Trivial name:   
SeqCoverage [median]: 10.80  
Mass: 17.48 kDa  
  
 

| Sequence | MissedCleavage | Mass [Da] | wtIntensity | muIntensity | Score |
| --- | --- | --- | --- | --- | --- |
| MFDKLK | 1 | 780.42 | 0.00 | 408.14 | 48.12 |
| QAVGLQEQEEK | 0 | 1257.62 | 0.00 | 562.72 | 122.96 |
|  |  |  | 0.0 | 970.9 |  |
